# Small-Angle Neutron Scattering for Studying Lipid Bilayer Membranes

**DOI:** 10.3390/biom12111591

**Published:** 2022-10-29

**Authors:** William T. Heller

**Affiliations:** Neutron Scattering Division, Oak Ridge National Laboratory, Oak Ridge, TN 37831, USA; hellerwt@ornl.gov; Tel.: +1-865-241-0093

**Keywords:** small-angle neutron scattering, contrast variation, deuterium labeling

## Abstract

**Featured Application:**

**This manuscript has been authored by UT-Battelle, LLC under Contract DE-AC05-00OR22725 with the U.S. Department of Energy (DOE). The U.S. government retains and the publisher, by accepting the article for publication, acknowledges that the US government retains a nonexclusive, paid-up, irrevocable, worldwide license to publish or reproduce the published form of this manuscript, or allow others to do so, for U.S. government purposes.**

**Abstract:**

Small-angle neutron scattering (SANS) is a powerful tool for studying biological membranes and model lipid bilayer membranes. The length scales probed by SANS, being from 1 nm to over 100 nm, are well-matched to the relevant length scales of the bilayer, particularly when it is in the form of a vesicle. However, it is the ability of SANS to differentiate between isotopes of hydrogen as well as the availability of deuterium labeled lipids that truly enable SANS to reveal details of membranes that are not accessible with the use of other techniques, such as small-angle X-ray scattering. In this work, an overview of the use of SANS for studying unilamellar lipid bilayer vesicles is presented. The technique is briefly presented, and the power of selective deuteration and contrast variation methods is discussed. Approaches to modeling SANS data from unilamellar lipid bilayer vesicles are presented. Finally, recent examples are discussed. While the emphasis is on studies of unilamellar vesicles, examples of the use of SANS to study intact cells are also presented.

## 1. Introduction

**Figure 1:**
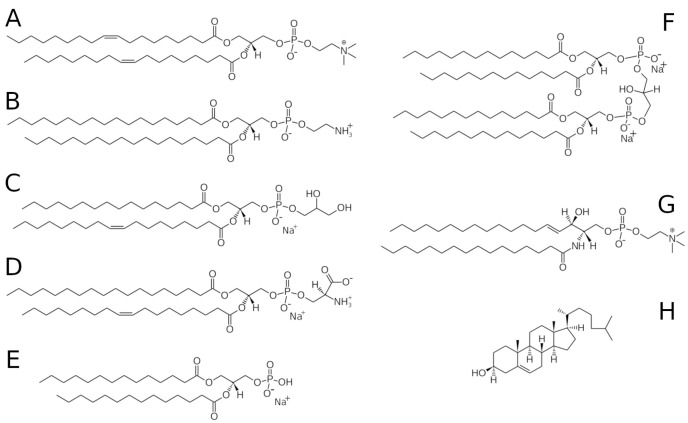
Chemical structures of (**A**) DOPC, (**B**) DSPE, (**C**) POPG, (**D**) SOPS, (**E**) DMPA, (**F**) cardiolipin, (**G**) sphingomyelin, and (**H**) cholesterol.

Biological membranes are composed of a wide variety of lipids and proteins and are integral to cellular function. Eukaryotic membranes contain a high fraction of PC and PE lipids (a list of abbreviations is provided immediately prior to the References), along with other phospholipids, cholesterol, sphingomyelin, and other spingolipids [1]. In contrast, bacterial membranes tend to be richer in PG and PE lipids, although their composition in different species can vary considerably [2,3]. Example lipids are shown in Figure 1. The complex mixture of lipids and proteins assembles into a well-defined and selectively permeable boundary between the interior and exterior of the cell and between structures within the cell. The membrane structure is not uniform across its thickness. The inner and outer leaflets of the membrane differ in composition [4], which is actively maintained by the cell [5]. Additionally, the structure of the membrane is not uniform within the plane of the membrane [6]. The interaction between the constituents of the bilayer provides the membrane with structure and a well-defined function, and no component works in isolation. The complexity of the membrane as well as the large range of length and time scales involved in its structure and function present a challenge to researchers who wish to understand it.

Multiple biophysical characterization techniques are required to understand the structure and function of biomembranes, because no single experimental technique is able to provide all of the required information. For example, protein conformation in the membrane environment can be probed by circular dichroism spectroscopy [7,8], solid-state magic-angle-spinning NMR [9], Fourier transform infrared spectroscopy [10], and fluorescence spectroscopy [11]. Structures in the membrane can be visualized at micron length scales by microscopy [12,13] and can be inferred from fluorescence spectroscopy [14]. NMR spectroscopy can be used to determine lipid acyl chain order parameters [15], and it can be used to determine the lipid bilayer structure [16].

X-ray and neutron scattering techniques are extensively used to determine the biomembrane structure. While diffraction can provide atomic-resolution information, SANS and SAXS are powerful tools for investigating structures in materials that lack crystallographic order at length scales from 10 Å to well over 1000 Å. Reflectometry is also a powerful technique for the study of biomembrane structure. The most detailed information comes from using highly oriented samples to perform lamellar diffraction [17,18,19,20,21,22,23,24,25]. Reflectometry also uses planar samples, but the samples typically studied are a single lipid bilayer adsorbed onto a highly flat substrate [26,27,28,29,30,31]. Reflectometry probes length scales similar to those of SANS, and it can be used in conjunction with SANS [32]. Hydrated dispersions and multilamellar vesicles, which provide powder diffraction data, generally provide less detailed structural information [33,34,35]. However, this is not necessarily the case because the entire scattering curve, if it is of high enough quality, can be fit for high-quality structural information [36,37]. As a result, SAXS studies often employ multilamellar vesicles. SANS instruments are not well-suited to these studies due to their lower ability to resolve features in the signal [38].

SANS and SAXS are powerful tools for studying lipid bilayer membranes in solution when prepared as unilamellar vesicles. Unilamellar vesicles are attractive because they are structurally similar to cell membranes, they are conceptually simple, and they are always in a fully hydrated state. They are also relatively easy to prepare using well-established methods such as rehydration from dried lipid films [39,40], extrusion [41,42], sonication [43,44], and electroformation [45]. Literature describing the difficulties that can be encountered during the preparation of unilamellar vesicles also exists [46,47,48]. As a result, experiments utilizing SANS and SAXS to investigate lipid bilayer membranes are readily undertaken by researchers from different backgrounds.

Here, a review of SANS for investigating lipid bilayer membranes is presented, with a particular focus on studies of unilamellar lipid bilayer vesicles. In addition to providing a brief overview of small-angle scattering, contrast variation methods for studying membranes are presented. Approaches to SANS data analysis for these systems are presented as well, which may also be applied to SAXS data. Finally, recent examples of the use of SANS to study lipid bilayer membranes are provided, with some examples of SANS being used in studies of intact, living biological membranes that help to show how studies of model systems can expand into studies of natural systems.

## 2. Small-Angle Neutron Scattering

**Figure 2:**
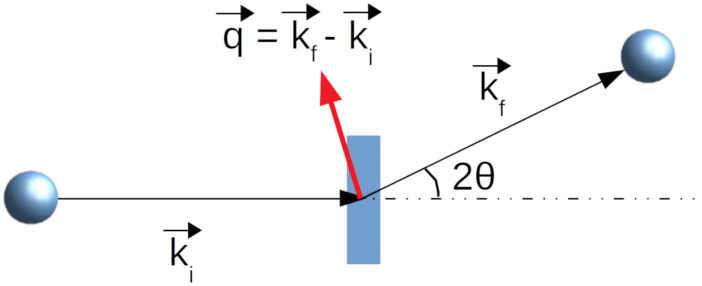
Definition of the scattering vector $\vec{q}$, which is shown in red.

Consider neutrons with wavelength λ, which interact with a sample and scatter from it, as shown schematically in Figure 2. The neutrons are incident on the sample with momentum $\vec{k_{i}}$ and exit the sample with momentum $\vec{k_{f}}$, which is an angle 2θ from the incident neutron beam direction. Note that only elastic scattering is being considered, and thus $∥\vec{k_{i}}∥=∥\vec{k_{f}}∥=2\pi /\lambda$. SANS data are measured as a function of the momentum transfer $\vec{q}=\vec{k_{f}}-\vec{k_{i}}$, which has a magnitude of $∥\vec{q}∥=\left(4\pi /\lambda \right)sin\left(\theta \right)$ and is referred to as *q*. It is related to the length scale *d* that is probed through d=2π/q. By measuring at small angles, i.e., small *q*, structures at large length scales relative to the neutron wavelength can be studied.

When neutrons interact with the atoms in the sample, they radiate out as circular waves (recall the wave–particle duality) that are centered on each atom. Small-angle scattering results from the interference of these waves. The measured small-angle scattering intensity of a material, regardless of whether neutrons or X-rays are used, is given by Equation (1) [49,50].

(1) $$I\left(\vec{q}\right)=|\int_{V}\rho \left(\vec{r}\right)e^{-i\vec{q}\cdot \vec{r}}d^{3}r{|}^{2}$$

Here, $\rho (\vec{r})$ is the density of the material property that interacts with the probe. For neutrons, the density of interest is the neutron scattering length density, referred to as the SLD, which is discussed in Section 3. The integral is taken over the volume of the sample exposed to the incident beam *V*.

When applied to dilute solutions, such as lipid bilayer vesicles in water, Equation (1) can be re-written as Equation (2) [49,50].

(2) $$I\left(q\right)=n_{p}\langle |\int_{V_{p}}\left[\rho \left(\vec{r}\right)-\rho_{s}\right]e^{-i\vec{q}\cdot \vec{r}}d^{3}r{|}^{2}\rangle$$

In Equation (2), $\rho (\vec{r})$ is the SLD inside the particle in solution, while $\rho_{s}$ is the SLD of the solvent. The integral is now taken over the volume of the particle $V_{p}$. The 〈〉 denotes averaging over all orientations of the particle with respect to the incident beam. Equation (2) can be alternatively expressed according to Equation (3).

(3) $$I\left(q\right)=n_{p}|F\left(q\right){|}^{2}S\left(q\right)$$

F(q) is referred to as the form factor, and S(q) is the structure factor. A lot of F(q)s are analytical functions [51], which is very helpful when performing data analysis. If there are no interparticle interactions, S(q)=1.

## 3. Contrast Variation

SANS has advantages that make it excellently suited for the study of membranes. The low-energy neutrons used for SANS do not cause radiation damage to most samples, which cannot be said for the X-rays used in SAXS experiments. However, the unique sensitivity of neutrons to light elements and the isotopes of elements is their greatest advantage. Unlike X-rays, neutrons interact with most elements on the periodic table as well as with their isotopes, with a comparable strength that is represented by the coherent neutron scattering length $b_{coh}$, often simply referred to as the neutron scattering length. The $b_{coh}$ for the elements and several of their isotopes were tabulated by Sears [52], and those of interest for studies of membranes are presented in Table 1. Note that not only does ${}^{1}$H have a significantly different neutron scattering length as compared to ${}^{2}$H, but the sign of $b_{coh}$ also differs. ${}^{1}$H is one of the few elements that has a negative $b_{coh}$. The large difference between the $b_{coh}$ of ${}^{1}$H and ${}^{2}$H makes contrast variation methods for biological materials relatively easy [53].

Contrast variation leverages the difference in scattering length density of different compounds in a structure, such as lipids, nucleic acids, and proteins, as well as the ability to label these materials with ${}^{2}$H to highlight specific ones. The existence of contrast between materials in the sample means there are materials with different SLDs in the sample, and this makes it possible to differentiate portions of structures within the intact whole. The difference in SLD between two materials is often simply referred to as the contrast. Then, one varies the relative contrast between the parts of a structure by using different mixtures of H${}_{2}$O and ${}^{2}$H${}_{2}$O as the solvent.

To calculate the SLD, ρ, of a molecule, one needs to know its atomic and isotopic composition and its molecular volume *V*. Then, the SLD can be calculated using Equation (4). The summation is over all *i* atoms in the molecule.

(4) $$\rho =\frac{\sum_{i}b_{i,coh}}{V}$$

**Figure 3:**
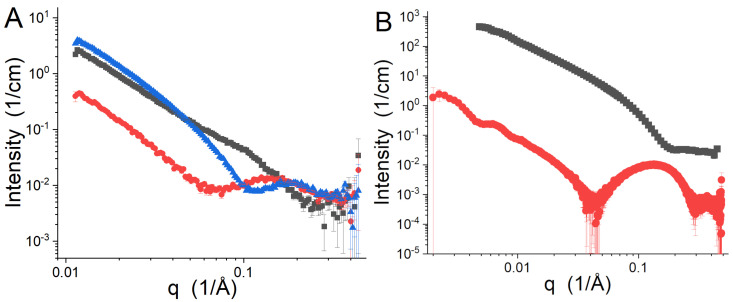
(**A**) SANS data from 7:3 ${}^{2}$H${}_{54}$-DMPC:DMPE at 55 ${}^{\circ }$C in 50% ${}^{2}$H${}_{2}$O (black squares), 70% ${}^{2}$H${}_{2}$O (red circles), and 90% ${}^{2}$H${}_{2}$O (blue triangles). The data were collected using the EQ-SANS instrument [88] and have not been previously published. (**B**) SANS (black squares) and SAXS data (red circles) of 7:3 DMPC:DMPG vesicles in ${}^{2}$H${}_{2}$O. The SANS data were taken from a previously published study [89], while the SAXS data were collected from Sector 12-ID-B of the Advanced Photon Source of Argonne National Laboratory and have not been previously published.

It is necessary to account for hydrogen exchange with certain chemical groups, such as hydroxyl groups, when samples are in mixtures of H${}_{2}$O and ${}^{2}$H${}_{2}$O. The molecular volume can be calculated from the molecular weight and density, if these are known. The volumes of various lipids as well as many head groups have been presented in a broad body of literature [19,54,55,56,57,58,59,60,61,62,63,64,65,66,67,68,69,70,71,72,73,74,75,76,77,78,79,80,81,82,83]. It is important to note that not all of the published values for a given lipid, HG, or acyl chain agree. Temperature-dependent lipid volumes have also been measured [74,76,82,83,84]. Several example lipid HGs, acyl chains, and other example compounds encountered in studies of biomembranes are presented in Table 2. The convention for separating the head group from the non-polar groups of the lipids is presented in Figure 3 of the review by Marsh [80]. The SLDs of proteins, DNA, and RNA can be readily calculated from the sequence and the molecular volume. Information about the amino acid and nucleic acid volumes, exchangeable protons, and scattering lengths in H${}_{2}$O and ${}^{2}$H${}_{2}$O are provided in Table 2 of the excellent review by Jacrot [85] and in the more recent comprehensive review by Breyton and coworkers [86]. The online activation calculator provided by the National Institute of Standards and Technology’s Center for Neutron Research can also provide the SLDs of proteins, RNA, and DNA [87]. The peptide melittin is also presented in Table 2 as an example.

In lipid bilayer membranes containing proteins or more than a single species of lipid, contrast variation methods make it possible to observe the protein in the vesicle or to see how the various lipid species are distributed. Lipids, proteins, and nucleic acids have intrinsic contrast without deuterium labeling [53]. To obtain a sufficient contrast between different lipids, deuterium-labeled lipids are needed. Fortunately, a wide variety of deuterium-labeled lipids, including those that are fully deuterated and those with specific chemical groups deuterated, are available for purchase from vendors. However, typically, only saturated acyl chains can be acquired in deuterated form from vendors. When studying lipid bilayer vesicles composed of a single lipid, one need not resort to contrast variation methods. SAXS is sufficient because there is electron density contrast between the lipid HGs and the acyl chains. SAXS data can also serve as an additional contrast point in a contrast variation study.

An example of contrast variation and selective deuterium labeling can be seen in Figure 3. Figure 3A presents SANS data from vesicles made of ${}^{2}$H${}_{54}$-DMPC (i.e., the acyl chains are fully deuterated) at a 7:3 molar ratio and DMPE. The SLDs of ${}^{2}$H${}_{54}$-DMPC and DMPE are 5.394 $\times \;10^{-6}$ Å${}^{-2}$ and 0.323 $\times \;10^{-6}$ Å${}^{-2}$, respectively, while the average SLD of the acyl chains of this composition is 4.673 $\times \;10^{-6}$ Å${}^{-2}$. The samples were measured in three different mixtures of H${}_{2}$O and ${}^{2}$H${}_{2}$O: 50%, 70%, and 90% ${}^{2}$H${}_{2}$O, which have SLDs of 2.912 $\times \;10^{-6}$ Å${}^{-2}$, 4.301 $\times \;10^{-6}$ Å${}^{-2}$, and 5.690 $\times \;10^{-6}$ Å${}^{-2}$, respectively. The 50% ${}^{2}$H${}_{2}$O SANS data result from the interplay of competing positive and negative contrasts. The 70% ${}^{2}$H${}_{2}$O SANS data are dominated by the scattering from the HGs because the average SLD of the chains are nearly the same as that of the solvent. The 90% ${}^{2}$H${}_{2}$O SANS data are dominated by the distribution of the DMPE in the vesicles.

In Figure 3B, SANS and SAXS data collected from vesicles made of a 7:3 molar mixture of DMPC and DMPG in ${}^{2}$H${}_{2}$O are presented. The SANS data are from a previous study [89]. The hydrogenated lipids in ${}^{2}$H${}_{2}$O have excellent contrast for SANS, but the details are lost because the contrast between the solvent and both the acyl chains and the HGs have the same sign and are relatively large, resulting in a less feature-rich data set. The contrast in the SAXS data set comes from the electron density of water being intermediate to those of the electron-rich HGs and acyl chains. The much better *q*-resolution (i.e., the uncertainty in *q* arising from the instrument) provided by SAXS instruments as compared to SANS instruments can also be seen in the sharper features in the SAXS data relative to those of the 50% ${}^{2}$H${}_{2}$O SANS data in Figure 3A, even though the interplay of the contrasts in the samples are very similar.

## 4. Data Analysis

SANS data analysis and modeling for unilamellar lipid bilayer vesicles are reasonably straightforward. A variety of approaches that provide different levels of detail are used. A modified form of the Guinier analysis can be used to determine the bilayer thickness. Simple core–shell models are frequently used, but improved approaches that provide even more detailed structural information are available. Molecular dynamics simulations can also play a role. Here, a brief overview of data analysis methods for the interpretation of SANS data from unilamellar lipid bilayer vesicles is presented.

It is possible to determine the thickness of the bilayer by fitting the SANS data with the use of the Kratky–Porod approximation [90,91,92,93,94,95,96], which is similar to the Guinier analysis [50]. If the thickness of the vesicle, $T_{v}$, is much smaller than the radius of the vesicle, $R_{v}$, then the data, I(q), can be expressed as $I_{KP}$ using the approximation shown in Equation (5).

(5) $$I_{KP}\left(q\right)\approx \frac{I(q=0)}{q^{2}}e^{-q^{2}R_{T}^{2}}$$

**Figure 4:**
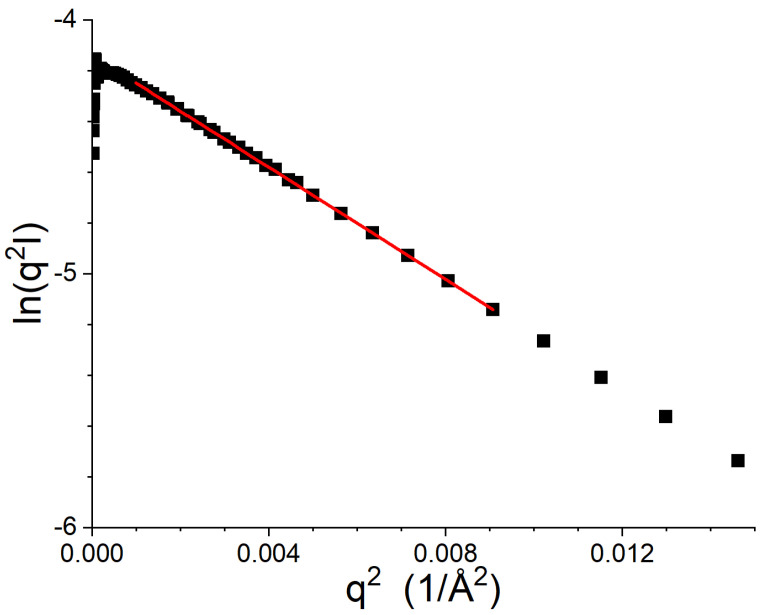
Example of fitting data using Equation (5). The SANS data from Figure 3B are the black squares, while the results of the fit using Equation (5) are shown as the solid red line. $T_{v}$ is 36.4 Å. The low Q portion of the data was not fit because the oscillations resulting from the form factor of the entire vesicle are visible.

$R_{T}$ is the radius of gyration of the thickness, and $T_{v}=R_{T}\sqrt{12}$. $R_{T}$ is determined by fitting a straight line to $Ln(q^{2}I\left(q\right))$ vs. $q^{2}$. The approximation is only valid for a region of *q* that is bound by structural features of the vesicles. More specifically, data fitting using the approximation shown in Equation (5) must be restricted to $1/R_{v}\leq q\leq 1/R_{T}$ [95]. Alternatively, the linear fit can be restricted to $2\pi /\sqrt{S_{v}}\leq q\leq 1/R_{T}$, where $S_{v}=4\pi R_{v}^{2}$ is the surface area of the vesicle [90,91,92]. An example of this kind of analysis is shown in Figure 4. When contrast variation methods are applied, additional details about the structure of the bilayer can be extracted from this analysis [90,91,92,93,94,95,96].

The most straightforward approach for modeling the structure of the vesicle is through the use of a core–shell model to fit the data [51]. The model intensity profile is shown in Equation (6) [51].

(6) $$I\left(q\right)=A{\left[\sum_{i=1}^{N}\left(\left(\rho_{i+1}-\rho_{i}\right)\frac{4\pi R_{i}^{3}}{3}\right)\left(\frac{3(\sin qR_{i}-qR_{i}\cos qR_{i})}{{\left(qR_{i}\right)}^{3}}\right)\right]}^{2}+B$$

In the equation, $\rho_{1}$ is the SLD of the core, which is assumed to be the same as the solvent ($\rho_{N+1}$), while the $\rho_{i}$ represents the SLDs of the various shells in the model. The radii of the various shells, starting from the radius of the core, are represented by $R_{i}$. If the thickness of a shell in the model is $T_{i}$, then $R_{i+1}$ = $R_{i}+T_{i}$. The radius of the core of the vesicle, which is $R_{1}$, may be polydisperse, but the radii of the layers are normally assumed to have a single value. There is always one more term in the summation than there are shells in the model. *A* and *B* are a multiplicative constant and an additive baseline, respectively. *A* is related to the sum of the products of the SLDs and volumes of the shells in the model, and the concentration of the vesicles. It is often easier to simply allow it to be a free parameter. *B* accounts for the incoherent scattering from hydrogen, but it also accounts for background subtraction artifacts during data reduction.

**Figure 5:**
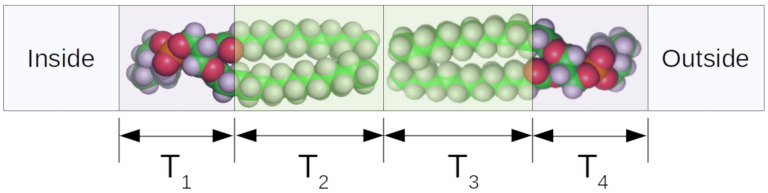
Schematic of a simple four-shell model showing how the four layers correspond to regions of the bilayer. The layer thicknesses are represented by $T_{i}$. The lipid was rendered using PyMOL 2.5.2 [97].

The number of shells to be used depends on the complexity of the bilayer structure. If three shells are used, then they represent an inner HG region, the acyl chain region, and the outer HG region. When contrast variation methods are used in samples containing mixtures of lipids, such as for the samples shown in Figure 3A, four shells are reasonable because the hydrogenated and deuterated lipids may not be equally distributed between the leaflets of the bilayer. The four shells represent the inner HG, the inner acyl chain region, the outer acyl chain region, and the outer HG, shown schematically in Figure 5.

A variety of approaches exist for determining the $T_{i}$ and $\rho_{i}$ of the various shells in the model. The values of $T_{i}$ and $\rho_{i}$ are allowed to be free parameters, but they may be constrained by the known physical parameters and the composition of the samples. Modeling SANS data from lipid bilayer vesicles in this way is relatively simple. No new software must be developed for the data analysis because a suitable model is provided in the SasView software package [98]. However, relating the resulting SLDs of the layers to their composition is not trivial.

An extension of this modeling approach is carried out by calculating the SLDs of the layers in the model from the known composition of the samples [99,100,101,102]. Consider a vesicle made from an undeuterated lipid that has a volume of $V_{L1}$ and HG and acyl chain volumes of $V_{HG1}$ and $V_{AC1}$, respectively, and a deuterium-labeled lipid with a volume of $V_{L2}$ and HG and acyl chain volumes of $V_{HG2}$ and $V_{AC2}$, respectively. Assume that the compositions of the inner and outer leaflets of the lipid bilayer are identical; hence, using a three-shell model is appropriate. Let the SLDs of the HGs and acyl chains of the lipids be $\rho_{HG1}$, $\rho_{AC1}$, $\rho_{HG2}$, and $\rho_{AC2}$. Denote the molar fraction of the unlabeled lipid with *f*, and let the number of waters per lipid associated with the inner and outer HG regions of the bilayer be $N_{w,in}$ and $N_{w,out}$, respectively. The SLD of water is denoted by $\rho_{w}$, and the volume of a water molecule is $V_{w}$. The SLDs of the three layers in the model are calculated using Equations (7)–(9).

(7) $$\rho_{HG,in}=\frac{f\rho_{HG1}V_{HG1}+\left(1-f\right)\rho_{HG2}V_{HG2}+\rho_{w}N_{w,in}V_{w}}{fV_{HG1}+\left(1-f\right)V_{HG2}+N_{w,in}V_{w}}.$$

(8) $$\rho_{AC}=\frac{f\rho_{AC1}V_{AC1}+\left(1-f\right)\rho_{AC2}V_{AC2}}{fV_{AC1}+\left(1-f\right)V_{AC2}}.$$

(9) $$\rho_{HG,out}=\frac{f\rho_{HG1}V_{HG1}+\left(1-f\right)\rho_{HG2}V_{HG2}+\rho_{w}N_{w,out}V_{w}}{fV_{HG1}+\left(1-f\right)V_{HG2}+N_{w,out}V_{w}}.$$

Note that it is not necessary to include a factor of 2 in Equation (8) because both the numerator and denominator would contain the factor of 2.

**Figure 6:**
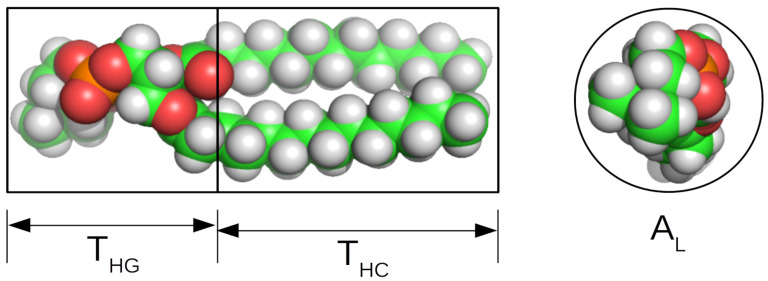
Schematic of parameters used in the self-consistent slab model showing how the lipid layer thickness might be divided into HG and acyl chain regions (**left**), and the area per lipid used to determine $D_{HC}$ and the number of waters associated with the lipid HG (**right**). The lipid was rendered using PyMOL 2.5.2 [97].

In light of the wealth of information that is available regarding lipid volumes and the volumes of acyl chains and HGs [19,54,55,56,57,58,59,60,61,62,63,64,65,66,67,68,69,70,71,72,73,74,75,76,77,78,79,80,81,82,83], it is possible to provide additional constraints on the structure of the lipid bilayer that is represented by the shells; this is termed the self-consistent slab model [103,104,105]. Recently, a version of the model was made available by Tan and coworkers [106] for use in the *Sasview* data analysis software package [98]. The self-consistent slab model shares features of other core–shell models that use the composition to determine the SLDs of the various layers in the model, but the model also uses known volumes for components of the bilayer to define the layer thicknesses and to provide a physically realistic structure. Here, one way of defining a self-consistent model is described using the schematic in Figure 6.

Assume that the bilayer has a single component. The area per lipid, $A_{L}$, and the volume of the acyl chains, $V_{HC}$, are used to determine the thickness of the acyl chain region, $T_{HC}$, according to Equation (10). The model described here does not take the curvature of the vesicle into account, as in other self-consistent models [103,104,105,106], because it assumes that $A_{L}$ is constant along the entire lipid molecule.

(10) $$T_{HC}=\frac{V_{HC}}{A_{L}}$$

The thickness of the HG region of the bilayer, $T_{HG}$, may be a free parameter if it is constrained such that $A_{L}T_{HG}$ cannot be smaller than the volume of the lipid HG, $V_{HG}$, or that it can be fixed [82,107,108], which is performed here. Alternatively, the model can be parameterized using the number of waters associated with the HG, $N_{w}$, which is then used to determine $T_{HG}$ [106]. Then, the thicknesses of the layers are given in Equation (11).

(11) $$T_{1}=T_{HG};\;\;T_{2}=2T_{HC};\;\;T_{3}=T_{HG}$$

$A_{L}$ also determines the number of waters associated with the HGs in the model here by using Equation (12).

(12) $$N_{w}=\frac{A_{L}T_{HG}-V_{HG}}{V_{w}}$$

The SLDs of the layers are calculated from the sample composition using Equations (7)–(9).

As the desire for more detailed structural information about the structure of lipid bilayers has increased, more detailed structural models have been developed. A notable result was the scattering density profile or SDP model [108,109]. Analytical functions are used to represent the position distributions of various chemical groups in the bilayer structure [109,110]. SDP models use constraints on the volumes of the groups that the lipid structure is divided into to ensure a physically realistic model. Fogarty and coworkers also expanded the modeling approach to an atomistic level of detail [111]. An online SDP modeling tool that allows anyone to fit their data using the approach was recently made available [112].

MD simulations can also be used for relating small-angle scattering data from lipid bilayer membranes to the membrane structure. Programs such as *n*Moldyn [113] or Sassena [114], which calculate X-ray and neutron scattering intensities directly from an MD trajectory, will not be discussed. Instead, methods more suitable for determining the transbilayer SLD profile are discussed here. Early work used simulations to calculate diffraction form factors, subsequently using the data to refine simulation parameters [115]. While the interpretation of diffraction data continued to be the main focus [110,116,117], the techniques later expanded to include SANS and SAXS [82,83,103,108,109,118,119,120,121]. MD simulations are less commonly used than the modeling methods described above because they are computationally intensive and require an MD expert. The position distributions of the various chemical groups in the bilayer can be determined directly from the simulation. Then, these distributions are used to calculate the bilayer SLD profile, which is subsequently used to calculate the SANS intensity profile that is compared with the measured data. Simulating an entire vesicle at an atomic resolution is prohibitive, but it can be accomplished using coarse-grained simulation methods such as the MARTINI approach [122]. Carillo and coworkers also developed a coarse-grained simulation method for modeling neutron scattering data [123].

Analyzing or modeling SANS data from vesicles that are not uniform in the plane of the bilayer—such as when domains form in a system that has been made using multiple lipids and when selective deuteration is employed—presents different challenges. System-specific modeling approaches are often required. When no obvious lateral domains are visible in the data, but there is evidence of two thicknesses in the same vesicle, then data can be modeled using an additive superposition of two bilayer models [100,124,125]. The Kratky–Porod approach to fitting data can reveal the presence of domains when contrast variation methods are utilized [96]. If a picture of the structures formed is desired, more complex models for the vesicle that account for the domains and the correlations between them are required. For example, Heberle and coworkers used a Monte Carlo approach to simulate the SANS data from vesicles that possessed large domains [126]. The models accurately reproduced the impact of the large domains on the SANS data and revealed how the domains depended on the composition of the vesicles and the temperature.

It is important to note that one of the challenges that faces modeling for SAS data analysis is the over-fitting of data due to limited information content [127,128]. It is possible to have more than one model fit the data equally well, particularly when too many free parameters are used. For example, a four-shell model, in which the layer thicknesses and SLDs are allowed to be free parameters, has ten free parameters (see Equation (6)) or more if the core radius and layers are polydisperse and the widths of the distributions are not fixed. It is generally preferable to use as few free parameters as possible. Self-consistent slab models and the SDP model help to reduce the number of free parameters required during fitting. Using molecular dynamics simulations to determine the SLD profile across the membrane also constrains the material spatial distributions. Laterally inhomogeneous systems may require several free parameters during fitting, and care must be taken to ensure that the results are robust.

## 5. Examples

Many researchers have employed SANS to study the structure of lipid bilayer vesicles. It has been used as a tool for understanding membrane structure, primarily in model systems containing a limited mix of lipid species, although more complex membranes have also been investigated. The technique has also found application in understanding the interaction of lipid bilayers with peptides and proteins, small molecules, and polymers. Intact cells and organisms have also been studied. Here, a survey of more recent examples taken from the large body of literature that employed SANS to study lipid bilayer membranes is presented.

### 5.1. SANS for Lipid Bilayer Structure Determination

SANS is a valuable tool for structural studies, both in concert with diffraction and by itself. Several different single-component PC lipid bilayers have been characterized by SANS, including the saturated DLPC [119,129], DMPC [91,95,119,129,130,131], DPPC [119,132,133,134,135], DSPC [119,129,136], and DPhPC [74,81]. PC lipids that have monounsaturated [74,119,137] and polyunsaturated [138] acyl chains have been studied as unilamellar vesicles using SANS. Various PG lipids [82], PE lipids [83], and PS lipids [120] have also been characterized. Cardiolipin bilayer membranes have been studied [139] as well as those formed by sphingomyelins [140], the GM3 ganglioside [141], and lipopolysaccharides extracted from cells [142,143]. More novel lipids have been studied using SANS, such as fully artificial ones [144] and siloxane phosphocholines [145]. SANS also provides the important membrane thickness needed for understanding the dynamics of lipid bilayers that have been measured by NSE [146,147,148,149,150,151,152,153,154], although the information reported about the structure may not be as detailed as what is found in a study dedicated to membrane structure.

More complex lipid mixtures composed of multiple phospholipids, or that incorporate cholesterol or sphingomyelin, have also been studied with the use of SANS. Cholesterol is an important constituent of mammalian cell membranes, and it is also medically important. The impact of cholesterol on the structure of phospholipid bilayers was the subject of several studies [84,155,156,157,158,159,160,161]. SANS has been used to determine the solubility limit of cholesterol in POPC and POPS unilamellar vesicles and found it to be quite different [162]. SANS and MD have been used to understand membranes made of ether lipids and cholesterol [163] as well as membranes from the stratum corneum [164]. The impact of ergosterol on DPPC membranes was determined using SANS [165]. DMPC/DMPG/DHPC bilayer vesicles that spontaneously form from a bicelle state have also been characterized [166,167,168]. A mixture of PC lipids with sphingomyelin was studied, which resulted in bilayers showing the significant interdigitation of the inner and outer leaflets of the membrane [169]. Mimics of bacterial membranes containing PE, PG, and various cardiolipins were probed by SANS [170]. DMPC-ceramide mixtures produced smaller vesicles than when only DMPC was present [171]. It is also possible to investigate vesicles in which the inner leaflet of the membrane does not have the same composition as the outer one [172,173,174,175,176].

**Figure 7:**
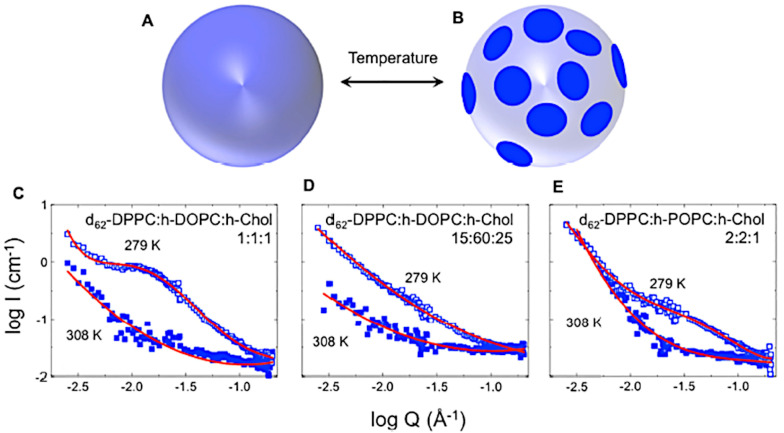
Temperature-dependent domain formation in multicomponent phospholipid bilayers. (**A**,**B**) represent a schematic of a transition from a laterally uniform vesicle to a state in which there are lateral domains with different SLDs. (**C**–**E**) show low temperatures (open squares) and high temperatures (solid squares) that reveal the growth of the domains. The figure is Figure 1 of reference [182] by Ahmadi et al. which was published under the Creative Commons Attribution License (https://creativecommons.org/licenses/by/4.0/, accessed on 22 September 2022). The authors of reference [182] hold the copyright for the figure.

SANS with contrast variation methods are excellent tools for investigating the formation of laterally separated domain structures in membranes such as rafts [6]. SANS has revealed lateral inhomogeneities in vesicles containing gangliosides [96,177], sphingomyelin [178], vesicles made of lipid mixtures that have very different acyl chain lengths [126], and simple phospholipid–cholesterol mixtures [179]. Domains have also been observed using SANS using contrast variation methods in lipid vesicles made of POPC and DPPC [180]. Ceramides were found to impact the stability of domains formed in mixtures containing DPPC, DOPC, and cholesterol [181]. Ahmadi and coworkers used SANS and QENS to characterize mixtures of DPPC, DOPC, and cholesterol and of DPPC, POPC, and cholesterol [182]. Both mixtures became phase-separated at a temperature below the gel phase transition of DPPC and displayed raft-like domains, shown schematically with the SANS data in Figure 7. Similarly, contrast-matching methods can be used to visualize domains in vesicles, as was carried out for a mixture of POPC and DSPC [183].

### 5.2. SANS for Studying Lipid Bilayer Interactions between Peptides and Proteins

**Figure 8:**
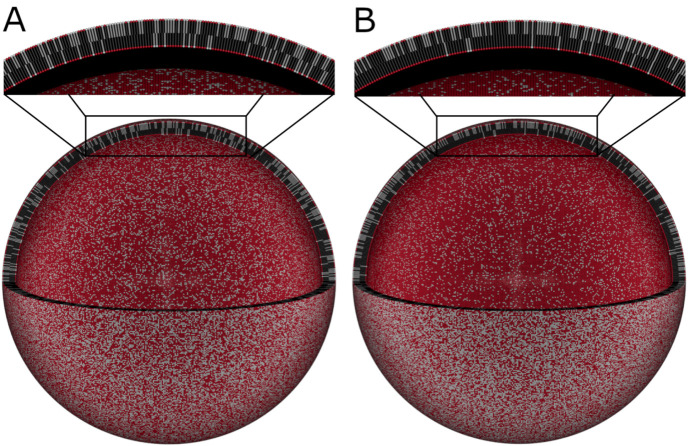
Schematic of the use of SANS with contrast variation and selective deuteration to determine how charged lipids respond to the peptide melittin [101]. DMPC:DMPS vesicles prepared at a molar ratio of 7:3 with and without the peptide melittin are shown with a wedge removed so that the inner leaflet of the bilayer can be seen. A zoomed-in view is also provided. (**A**) When no peptide is present, the outer leaflet of the bilayer is enhanced in charged lipids (the light structures) relative to neutral lipids (red and black structures). (**B**) When melittin is added at a peptide-to-lipid ratio of 1/200, further enhancement of the charged lipid content of the outer leaflet of the bilayer can be seen. The images in the figure used the results of the study in reference [101] and were created using the software developed for the table of contents graphic of the reference and the POV-Ray software version 3.7 [193]. The figure has not been published previously.

SANS with contrast variation is very useful for investigating the interaction of peptides and proteins with lipid bilayer membranes. The impact of the peptide on the structure of the bilayer can be determined, as well as how the peptide impacts the distribution of lipids if selective deterium labeling is employed. Several groups have used SANS in studies of peptides with lipid bilayers. Amyloid plaque-forming peptides such as the Alzheimer’s amyloid β peptide [184], α-synuclein [185], and the islet amyloid polypeptide [186] were investigated in lipid bilayer vesicles using SANS. The interaction and self-assembly of the peptides were studied. SANS and contrast variation methods are also excellent for studying the interaction of membrane-active and antimicrobial peptides with lipid bilayers because additional details about the interaction can be revealed when selective deuteration is employed [99,100,124,187,188,189]. It was possible to determine where indolicidin associates with vesicles [189] and how aurein 1.2 interacts with DMPC/DMPG vesicles [188]. The distribution of cholesterol was studied by Qian and coworkers when alamethicin [100] and melittin [124] interacted with vesicles made of DMPC and cholesterol, which indicated that both peptides disrupted how cholesterol was distributed in the vesicles. The movement of charged lipids between the inner and outer leaflets of the vesicles were also observed for alamethicin and melittin [99,187]. In Figure 8, the extent of charged lipid redistribution caused by melittin in DMPC/DMPS vesicles is shown schematically and demonstrates the sensitivity of contrast variation methods [187]. SANS can also be used to study the interaction of full-length proteins with lipid bilayer vesicles. Examples that employed contrast-matching techniques to minimize the scattering from the lipid include a study of cytochrome c oxidase [190] and the Bax/tBid system [191]. However, contrast matching is not required, as was demonstrated by Doktorova and coworkers in their study of the HIV-1 Gag protein [192].

**Figure 9:**
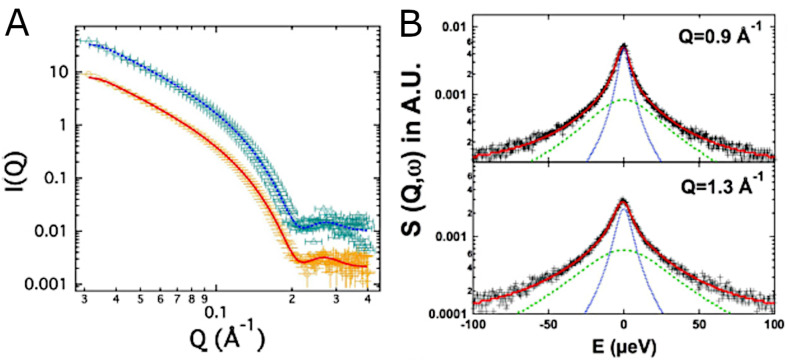
Impact of the amyloid β peptide on the structure and dynamics of DMPG membranes. (**A**) SANS data showing how the structure of the bilayer changes when peptide is absent from (orange) or present in (cyan) DMPG vesicles. (**B**) QENS data at two different *q*-values of DMPG vesicles with the amyloid β peptide. The figure is an adaptation of Figures 1a and 2a of reference [187] by Rai et al., which was published under the Creative Commons Attribution License (https://creativecommons.org/licenses/by/4.0/, accessed on 22 September 2022). The authors of reference [187] hold the copyright for the figure.

SANS is often used as a complementary tool in neutron spectroscopy studies of the impact of peptides on the dynamics of lipid bilayer membranes. QENS provides information about local dynamics in membranes, and it has been used with complementary SANS experiments to study the amyloid β peptide fragment (1–40) in DMPG membranes [187], which can be seen in Figure 9, as well as alamethicin and melittin in DMPC membranes [194]. Fusion peptides from the SARS-CoV-2 virus were found by QENS to make membranes more rigid [195]. At longer length- and time-scales, NSE and SANS are also valuable tools for understanding how peptides impact lipid bilayer membranes. In addition to revealing the impact of the SARS-CoV-2 fusion peptides on membranes [195], the impact of the HIV-1 fusion peptide has been studied [89], as has the amyloid β peptide [196]. Kelley and coworkers also determined how the antimicrobial peptides alamethicin and gramicidin slow down the collective dynamics of membranes, indicating that the membranes become stiffer in response to the peptides [197].

### 5.3. SANS for Lipid Bilayer Interactions with Other Materials

Several groups have investigated the interaction of lipid bilayers with compounds that are not proteins or peptides. The interaction of a variety of small molecules with membranes has been probed by SANS, including sugar [198,199,200], various alcohols produced during biofuel production [201], DMSO [92,202,203,204], glycerol [205], and carboranes [206]. Detergents [91,207,208,209], alkanes [210,211,212,213], and fatty acids [214] have also been studied. Anesthetics that interact directly with membranes were investigated in lipid bilayer membranes using SANS [215,216,217]. SANS improved the understanding of how vitamin E [218] and melatonin [219,220], both important for human health, interact with membranes. Compounds from food, some of which have medicinal effects, interacting with membranes have been studied using SANS, such as aescin from horse chestnut [221,222,223,224], glycyrrhizin from licorice [225], and epigallocatechin-3-gallate from green tea [226]. Furthermore, medicines have been studied. Painkillers, including ibuprofen [222,227] and acetominophen [228], interacting with lipid bilayers were probed with SANS. SANS was also used to study the interaction of the antifungal amphotericin B [229] and the antimicrobial HT61 [230] with lipid bilayer vesicles. Khadka and coworkers used SANS to characterize how the anticancer drug tamoxifen associates with lipid bilayers [231].

Polymers are of interest for drug delivery, in which the cell membrane plays an important role. They are also ubiquitous in daily life, and their interaction with membranes is of interest in a general sense. Poly(ethylene glycol)-modified lipids and their impact on vesicle structure were characterized using SANS [232], as were poly(ethylene glycol)-modified liposomes, which were studied for drug encapsulation and delivery [233] and for mRNA delivery [234]. The interaction of a poly(ethylene glycol)-polymer hybrid with lipid bilayer vesicles was investigated with the use of SANS [235]. By using contrast matching, domain formation in vesicles containing polymers was observed [236]. The accumulation of styrene oligomers in lipid bilayers was studied as a model for how plastic wastes interact with cell membranes [237]. The interaction of polythiophenes with model cell membranes was studied, which revealed that they associate with different regions of the bilayer depending on the specific chemistry of the terminal group of the polymer alkyl side chain [238]. The interaction of polymers that had been developed to have antibacterial action with lipid bilayer vesicles was investigated to learn how the polymer incorporates into the bilayer while still being able to function [239]. SANS revealed the oligomerization state of a polymer that selectively conducts protons, similar to some proteins, when incorporated into vesicles [240]. The location of monomers within the structure of the bilayer was also determined with SANS [241].

### 5.4. SANS for Studying the Kinetics of Lipid Bilayer Membranes

**Figure 10:**
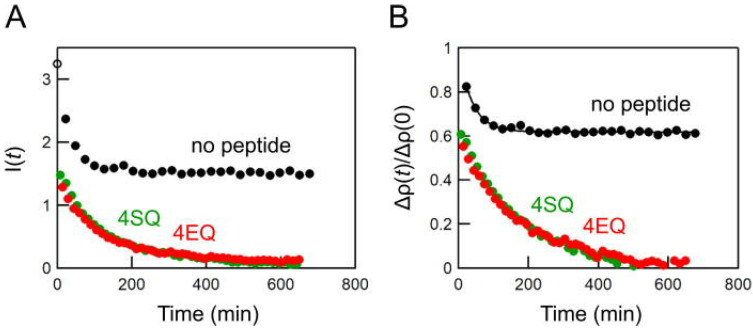
Peptide-enhanced exchange of lipids in vesicles [255]. (**A**) The integrated intensity as a function of time showing that two peptides engineered to scramble the lipids in the bilayer (4SQ and 4EQ) cause greater lipid exchange than when no peptide is present. (**B**) The decay in scattering contrast with time for the same system. The image is Figure 3 of reference [255] by Nakao et al., which was published under the Creative Commons Attribution License (https://creativecommons.org/licenses/by/4.0/, accessed on 22 September 2022). The authors of reference [255] hold the copyright for the figure.

The non-destructive nature of neutrons and the high flux of modern sources provide opportunities for studying kinetic processes in membranes. Such studies require contrast-matching methods and deuterium labeling. The movement of water across a model membrane over time was resolved with SANS [242]. SANS was also used to evaluate the transition of lipid nanodisks to unilamellar vesicles [243]. Simple curvature effects can facilitate lipid exchange [244], for example, and so can methanol [245]. The transport of cholesterol between POPC vesicles and between leaflets of a single vesicle was found by SANS to be slower than previously reported [246]. Later work revealed that cholesterol exchange happens more rapidly when unsaturated PC lipids are included in the vesicles [247], but it happens more slowly in POPS vesicles [248]. The kinetics of lipid exchange enabled by the SARS-CoV-2 fusion peptide was also probed using SANS [195]. The peptide-driven lipid exchange caused by melittin and alamethicin was also investigated [249]. The peptide indolicidin speeds up lipid exchange between vesicles while also making them grow [250], as do the antimicrobial peptides Aurein 1.2, LL-37, and Lacticin Q [251] Other peptides have a similar effect [252,253,254]. A series of peptides designed to scramble lipids in a bilayer was studied by Nakao and coworkers using SANS and contrast-matching methods [255]. By tracking the integrated intensity in a specific *q*-range, they were able to measure the rate of change in the neutron scattering contrast and therefore the lipid exchange. Examples of intensity decay curves are shown in Figure 10.

### 5.5. SANS of Intact Cell Membranes

SANS has also been used to study membranes in intact cells and organisms. One of the main advantages of using SANS to study living cells is the lack of radiation damage by neutrons that possess much lower energy than the X-ray photons used for SAXS experiments. However, applying contrast variation methods is more difficult due to the toxicity of ${}^{2}$H${}_{2}$O. Deuterium incorporation into plants also remains challenging [256]. Achieving the selective deuteration of components within cells in order to provide strong contrast against the remainder of the cell is not trivial. The structural information about the biomembrane contained in the data is also partially obscured by the other structures in the cell, which have common length scales. In spite of the challenges, SANS is a powerful tool for studying intact, living biological membranes, and examples of this follow.

**Figure 11:**
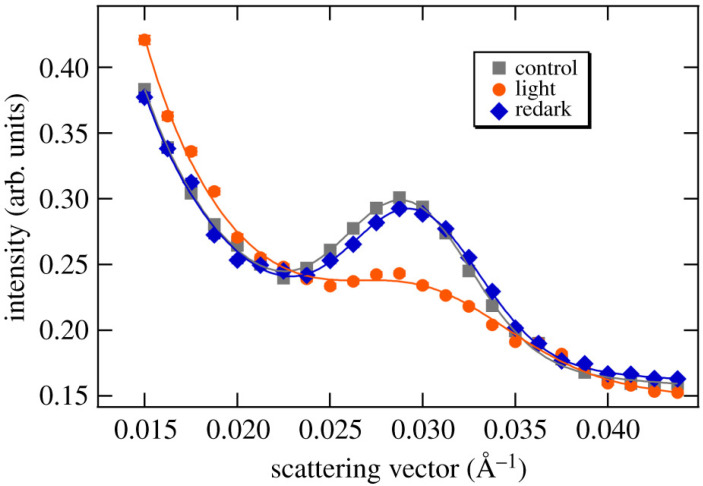
SANS data from a dark-adapted plant leaf showing the response of the thylakoid membranes to light cycling [266]. The image is Figure 1 of reference [266] by Ünnep et al., which was published under the Creative Commons Attribution License (https://creativecommons.org/licenses/by/4.0/, accessed on 22 September 2022). The authors of reference [266] hold the copyright for the figure.

One example of where SANS has been used to study intact cells is in investigations of the thylakoid membranes, which are part of the photosynthetic apparatus of plants [257,258,259,260,261,262,263,264,265,266,267]. Thylakoid membranes organize into stacks, and thus they give rise to diffraction features in SANS patterns that can be resolved from the remainder of the signal of the leaf of the plant or cell that is being measured. SANS studies have been performed on cyanobacteria [257,259,260,263,264], diatoms [258], algae [261,267], and higher plants [262,265,266]. The impact of environmental stressors, such as salt [267], selenium [265] and herbicide [264], on the structure of the membranes has also been studied. Cyanobacteria with mutations in the photosynthetic apparatus were studied using SANS to reveal the impact of the mutations on the structure of the membranes [259,260]. The response of the membranes to light has received considerable attention in various studies [257,258,259,262,264,266]. Ünnep and coworkers studied dark-adapted *M. deliciosa* (split-leaf philodendron) leaves and how they respond to exposure to light [266]. They observed a clear change in the thylakoid membrane signal after 8 min of light exposure, followed by 30 min in the dark, as can be seen in Figure 11.

**Figure 12:**
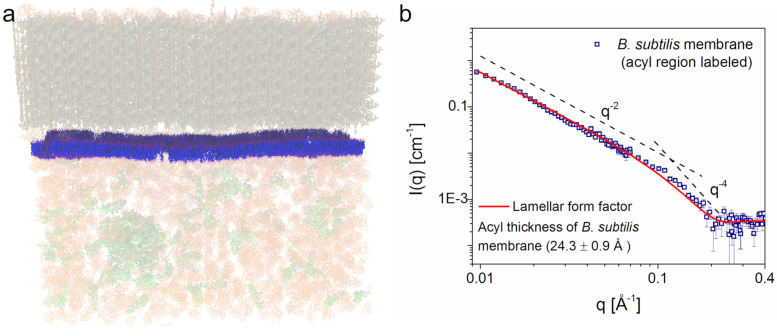
SANS with contrast matching applied to a living cell membrane [271]. (**a**) By feeding the cell deuterium-labeled fatty acids, it was possible to highlight the acyl core of the lipid bilayer. (**b**) The resulting SANS data could be fit for the thickness of the acyl chain region of the cell membrane. The image is Figure 3 of reference [271] by Nickels et al., which was published under the Creative Commons Attribution License (https://creativecommons.org/licenses/by/4.0/, accessed on 22 September 2022). The authors of reference [271] hold the copyright for the figure.

SANS measurements have revealed information about the structure of other cellular membranes as well. SANS revealed the repeat spacing of the membranes within mitochondria [268]. SANS with contrast variation and SAXS were used to develop a model of living *E. coli* cells, which provided information about the inner and outer membranes of the bacteria [269,270]. Nickels and coworkers used contrast-matching methods to study *B. subtilis* cellular membranes in vivo [271]. By feeding the bacteria with deuterated fatty acids, it was possible to measure the samples in a mixture of H${}_{2}$O and ${}^{2}$H${}_{2}$O, which minimized the scattering from everything in the cell, except for the acyl chain region of the cell membrane. Doing so allowed the team to measure the thickness of the cellular membrane, as can be seen in Figure 12, as well as to observe domains within its lateral structure [271].

## 6. Conclusions

SANS is a powerful tool for studying the structure of lipid bilayer membranes, and it will remain important in the future. When enhanced with contrast variation methods and selective deuterium labeling, it is possible to obtain information at the nanoscale about multi-component lipid mixtures, which is difficult, if not impossible, to to attain with the use of other experimental techniques. In addition, there are many opportunities to use these approaches in order to gain the insight needed to understand the complexity of cellular membranes. In particular, details about lipid distribution in samples made of more than one lipid are readily accessible, and the intrinsic contrast between proteins and lipids provides opportunities to study their interactions. Another area where future opportunities exist is in using SANS with contrast variation methods to investigate the structures and interactions of proteins that have been reconstituted into vesicles. In particular, using SANS and contrast variation methods would be a powerful tool for observing the oligomerization states of proteins or the complex formation, in the case of two or more different proteins, in lipid bilayer vesicles. The more native-like environment of the vesicle would allow for a more physically reasonable association to take place. In spite of the challenges intrinsic to selective deuterium labeling in living cells, the work of Nickels and coworkers demonstrates that such experiments can provide truly unique information about living cell membranes [271], and additional developments in this area have considerable potential for scientific impact.

A great deal can be learned using more traditional SANS data analysis approaches, which are readily accessible to both expert and novice users of the technique. However, not all of the more advanced approaches are necessarily accessible to the novice or casual user. Opportunities to improve the availability of such data analysis approaches exist. Furthermore, the continued development of more advanced modeling methods, such as those leveraging MD simulations, has excellent potential for revealing additional details when used in the analysis and interpretation of SANS data. The additional development of methods for modeling laterally inhomogeneous structures and making them widely accessible would also benefit the existing and potential user community. As is always the case with SAS data, care would need to be taken in order to avoid an over-interpretation of the data.

## Figures and Tables

**Table 1:** Coherent scattering lengths ($b_{coh}$) of isotopes of interest for biological membranes [52]. If no specific isotope is noted, the value provided is for the natural abundance of stable isotopes.

Isotope	$b_{\mathrm{coh}}$ (fm)
${}^{1}$H	−3.74
${}^{2}$H	6.67
C	6.65
N	9.36
O	5.81
P	5.13

**Table 2:** Example neutron SLDs encountered in SANS studies of biomembranes.

	Chemical or Group	ρ ($10^{-6}$ Å${}^{-2}$)
water		
	H${}_{2}$O	−0.560
	${}^{2}$H${}_{2}$O	6.384
		
phospholipid HGs		
	PC HG	1.877
	${}^{2}$H${}_{18}$-PC HG	7.736
	PE HG	2.483
	PA HG	2.494
	PG HG (in H${}_{2}$O)	2.382
	PG HG (in ${}^{2}$H${}_{2}$O)	3.076
	PS HG (in H${}_{2}$O)	3.466
	PS HG (in ${}^{2}$H${}_{2}$O)	3.893
		
acyl chains		
in phospholipids		
	lauroyl (12:0)	−0.386
	${}^{2}$H${}_{23}$-lauroyl (12:0)	6.755
	myristoyl (14:0)	−0.375
	${}^{2}$H${}_{27}$-myristoyl (14:0)	6.837
	palmitoyl (16:0)	−0.386
	${}^{2}$H${}_{31}$-palmitoyl (16:0)	6.557
	oleoyl (18:1c)	−0.386
		
other examples		
	cholesterol (in H${}_{2}$O)	0.216
	cholesterol (in ${}^{2}$H${}_{2}$O)	0.386
	melittin (in H${}_{2}$O)	1.444
	melittin (in ${}^{2}$H${}_{2}$O)	2.882

## Data Availability

Data available from the author on request.

## Abbreviations

The following abbreviations were used in this manuscript:

SANS	Small-angle neutron scattering
SAXS	Small-angle X-ray scattering
MD	Molecular dynamics
QENS	Quasi-elastic neutron scattering
NSE	Neutron spin echo spectroscopy
SLD	Neutron scattering length density
HG	Lipid head group
DHPC	1,2-dihexanoyl-sn-glycero-3-phosphocholine
DLPC	1,2-dilauroyl-sn-glycero-3-phosphocholine
DMPC	1,2-dimyristoyl-sn-glycero-3-phosphocholine
DPPC	1,2-dipalmitoyl-sn-glycero-3-phosphocholine
DSPC	1,2-distearoyl-sn-glycero-3-phosphocholine
DPhPC	1,2-diphytanyl-sn-glycero-3-phosphocholine
DMPG	1,2-dimyristoyl-sn-glycero-3-phospho-(1’-rac-glycerol) sodium salt
POPC	1-palmitoyl-2-oleoyl-sn-glycero-3-phosphocholine
POPG	1-palmitoyl-2-oleoyl-sn-glycero-3-phospho-(1’-rac-glycerol) sodium salt
DOPC	1,2-dioleoyl-sn-glycero-3-phosphocholine
POPS	1-palmitoyl-2-oleoyl-sn-glycero-3-phospho-L-serine sodium salt
DSPE	1,2-distearoyl-sn-glycero-3-phosphoethanolamine
POPS	1-stearoyl-2-oleoyl-sn-glycero-3-phospho-L-serine sodium salt
DMPA	1,2-dimyristoyl-sn-glycero-3-phosphate sodium salt
PC	Phosphatidylcholine lipid head group
PG	Phosphatidylglycerol lipid head group
PE	Phosphatidylethanolamine lipid head group
PS	Phosphatidylserine lipid head group
PA	Phosphatic acid lipid head group
